# Application of Needleman-Wunch Algorithm to identify mutation in DNA sequences of Corona virus

**DOI:** 10.1088/1742-6596/1218/1/012031

**Published:** 2019-05-01

**Authors:** Mohammad Isa Irawan, Imam Mukhlash, Abduh Rizky, Alfiana Ririsati Dewi

**Affiliations:** 1 Department of Mathematics, Faculty of Mathematics, Computation, And Data Sciences, Institut Teknologi Sepuluh Nopember Raya ITS Street, Keputih, Sukolilo, Surabaya 60111 Indonesiamii@its.ac.id; 2 Departement of Mathematics, University of Jember Jln. Kalimantan 37, Tegalboto, Jember 68121 Indonesia

## Abstract

—Corona virus is a virus capable of mutating very quickly and many other viruses that arise due to mutations of this virus. To find out the location of corona virus mutations of one type to another, DNA sequences can be aligned using the Needleman-Wunsch algorithm. Corona virus data was taken from Genbank National Center for Biotechnology Information from 1985-1992. The Needleman-Wunsch algorithm is a global alignment algorithm in which alignment is performed to all sequences with the complexity of O (mn) and is capable of producing optimal alignment. An important step in this reserach is the sequence alignment of two corona viruses using the Needleman-Wunsch algorithm. Second, identification of the location of mutations from the DNA of the virus. The result of this reserach is an alignment and the location of mutations of both sequences. The results of identification DNA mutation can be used to find out other viruses mutation corona virus as well as can be used in the field of health as a reference for the manufacture of drugs for corona virus mutation outcome.

## 1. Introduction

Computational biology is the field of science that focuses on the preparation of a mathematical model in solving and analyzing biological sequence problems. Computing in the field of biology is known as bioinformatics. Bioinformatics is the science of the combination of biological science and informatics for data storage, retrieval, data manipulation, and the distribution of information associated with biological macromolecules, such as DNA, RNA, and proteins. Bioinformatics is more often used for microbiological computing and focuses on analyzing biological sequences data.

DNA or Deoxyribonucleic Acid are biomolecules in the form of nucleic acids (found in the nucleus of cells), which function to store genetic information in an organism. Double-stranded DNA joins hydrogen bonds between bases in two strands. This base is Adenin (A), Cytosine (C), Guanine (G), and Thymine (T). DNA is a method that can prove whether an organism has a family relationship or not with other organisms. One of the introduction of an organism in bioinformatics is sequence alignment, which is the process of composing /aligning a sequence with one or more other sequences so that the sequence equations are clear or the level of similarity is obtained[1].

Mutation is a change in the genetic material of a creature that occurs unplanned, random, and is the basis for a heritable source of living organisms. From WHO data reported that as of May 31th, 2015 there were 1180 cases confirmed by laboratory that positive corona virus with 483 patients dying (40% mortality) [2].

Content from this work may be used under the terms of the Creative Commons Attribution 3.0 licence. Any further distribution of this work must maintain attribution to the author(s) and the title of the work, journal citation and DOI.
Published under licence by IOP Publishing Ltd

To find out whether this corona virus is a relative or not, a method is needed that can align the corona virus DNA with one another. These problems can be solved using dynamic programming. Dynamic programming in DNA sequence alignment has two types of techniques, namely global and local alignment. Some algorithms used in local alignment include Smith-Waterman, FASTA, BLAST, and many algorithms that are being developed. As for global alignment, the Needleman-Wunsch algorithm is still often used and also developed to be more efficient [3].

In this study, parallel alignment of DNA sequences is done globally because in this alignment, alignment is carried out from the end of the sequence to the other end of the sequence of the DNA character. The algorithm used in this study is the Needleman-Wunsch algorithm. This algorithm was originally created by Saul Needleman and Christian Wunsch in 1970 [4]. This algorithm is a dynamic programming implementation that is used to determine the level of similarity or the compatibility of two texts. The way this algorithm works is that DNA sequences are aligned by matching and shifting, so as to obtain the maximum global or overall level of similarity (Global Alignment) of the two DNA sequences with complexity O(mn). By looking at the results and the process of the Needleman-Wunsch algorithm in DNA alignment, this study discusses how the Needleman-Wunsch algorithm can be used to align the DNA sequences of the corona virus so that they can determine the presence of mutations in the corona virus. Corona virus DNA sequences data used is data in NCBI GenBank.

Several previous studies that underlie this research, among others, research conducted by Vijay Naidu and Ajit Narayanan [5] in 2016 the Needleman-Wunsch algorithm can be used to align two polymorphic malware viruses. Another study conducted by Mikhael Avner Malendes and Hendra Bunyamin [6] in 2017, concluded that the Needleman-Wunsch Algorithm had superior performance to align the sequence for both small and large data.

In this, research on sequence alignment on corona viruses using the needleman-wunsch algorithm is carried out which will then identify mutations in the sequence and the virus. The program implementation is done using the Python programming language with Anaconda software and Jupyter Notebook 5.0.0 application.

## 2. Fundamental Theory

### *2.1 Mutation*

Mutation is a change in structure in the genetic material of a creature that occurs randomly and is the basis for the source of a variety of living organisms that are heritable.

**Classification of Mutation**

There are 4 mutation classifications, namely [16]:

1. Type 1
   A mutation caused by changes in nucleotides, for example "a" changes to "g".
2. Type 2
   A mutation that occurs because there are parts of the nucleotide that change the order of its position, for example the "check" section changes the order to "guacc".
3. Type 3
   A mutation caused by the insertion of a new segment into the sequence, for example the insertion of "aa" in the middle of the "gguugg" segment will change the seggment to "gguaaugg".
4. Type 4
   A mutation that occurs due to the elimination of nucleotide segments in the sequence, for example the removal of nucleotide "ag" from the segment "acaguua" so that the segment changes to "acuua".

Because type 1 and type 2 mutations do not change the position of all nucleotides, these mutations are called substitution mutations. While type 3 and type 4 mutations are called transfer mutations because they can change the position of nucleotides.

*2.2 Sequence Alignment*

Sequence in bioinformatics can be described using the following notation:

$$A = (a_1, a_2, a_3, \dots, a_{n_a}) \quad B = (b_1, b_2, b_3, \dots, b_{n_b})$$
$$C = (c_1, c_2, c_3, \dots, c_{n_c}) \tag{1}$$

With A, B, C as sequence. $a_i, b_i, c_i$ states the basic units of the sequence in position to-i, where these elements are obtained from the set $V_q = \{0,1, \dots, q-1)$. Length of A, B, C is $n_a, n_b, n_c$.

Sequence alignment is the process of composing / aligning a sequence with one or more other sequences so that the sequence equations are clear or the level of similarity is obtained[1].

Here is an example of the alignment of two different short DNA sequences

```
A _ G _ C T A G A
|   |   | | | | |
A G G T C T A G A
```

Sign | states the existence of a match or match between the two sequences. DNA sequence alignment has two types of techniques, namely global and local alignment. Global alignment is the alignment performed for the whole sequence, some of the algorithms used in local alignment include Smith-Waterman, FASTA, BLAST, and many algorithms that are being developed. Whereas local alignment is alignment done to several or part of the sequence, the algorithm commonly used is the Needleman-Wunsch algorithm.

*2.3 Needleman-Wunsch Algorithm*

The Needleman-Wunsch algorithm is an implementation of dynamic programs (dynamic programming). The Needleman-Wunsch algorithm is used to determine the level of similarity or compatibility of two texts. This algorithm is also used to find alignments that have optimal values on global alignment in two sequences. This algorithm was created by Saul Needleman and Christian Wunsch in 1970 [7].

As for the steps to work on the Needleman-Wunsch algorithm is:

a. Matrix Initialization

For example sequences $A = a_1, a_2, \dots, a_n$ and $B = b_1, b_2, \dots, b_m$, then create a score matrix of size (n+1) x (m+1). Where n is the number of rows stating the length of the first sequence, and m is the number of columns stating the length of the second sequence. Then fill in the first row and the first column of the value matrix with the value of the gap penalty. Gap penalty is the value obtained when comparing the residues in a sequence with blank characters (gaps) in other sequences.

b. Charging Matrix

Suppose the value matrix is called the matrix S, then the formula for the elements of the matrix S is

$$S(i,j) = max \begin{cases} S(i-1, j-1) + s(a_i, b_j) \\ S(i-1, j) - d \\ S(i, j-1) - d \end{cases} \tag{2}$$

where:

- $S(i-1, j-1)$ = The matrix S element is diagonally left above
- $S(i, j-1)$ = The matrix S element on the left $S(i,j)$
- $S(i-1, j)$ = The matrix S element above $S(i,j)$
- $s(a_i, b_j)$ = the substitution matrix element in residue i in sequence a and residual j in sequence b
- $d$ = gap penalty in *virtual symbol*

With assumption gap linear model, is that

$$s(-, a) = s(a, -) = -d \text{ for } a \in Q \text{ where } d > 0 \tag{3}$$

IOP Conf. Series: Journal of Physics: Conf. Series **1218** (2019) 012031 doi:10.1088/1742-6596/1218/1/012031

then the value of the gap area with length L equals $-dL$. If the virtual symbol penalty score is d, then
$s(0,j) = -jxd, s(i,0) = -ixd, s(0,0) = 0$, and

$$s(a_i, b_j) = \begin{cases} match\ score, if\ a_i = b_j \\ mismatch\ score, if\ a_i \neq b_j \end{cases} \quad (4)$$

| | | $b_1$ | $b_2$ | $b_3$ | ... | $b_m$ |
|---|---|---|---|---|---|---|
| | $s(0,0)$ | $s(0,1)$ | $s(0,2)$ | $s(0,3)$ | ... | $s(0,m)$ |
| $a_1$ | $s(1,0)$ | $s(1,1)$ | $s(1,2)$ | $s(1,3)$ | ... | $s(1,m)$ |
| $a_2$ | $s(2,0)$ | $s(2,1)$ | $s(2,2)$ | $s(2,3)$ | ... | $s(2,m)$ |
| ... | ... | ... | | | | ... |
| $a_n$ | $s(n,0)$ | $s(n,1)$ | $s(n,2)$ | $s(n,3)$ | ... | $s(n,m)$ |

**Figure 1.** Substitution matrix of sequences A and B [8]

c. Traceback Step
After a score matrix of size (n + 1) x (m + 1) is fully filled, then the alignment score (the sum of all substitution values plus the sum of all gap penalties) is the maximum of two sequences is the value of the most element bottom right of the score matrix, that is $S(n+1, m+1) = s(n,m)$.

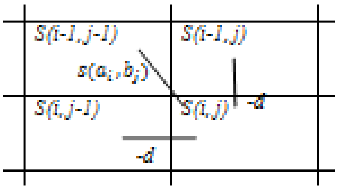

**Figure 2.** *Traceback Step* [9]

S(n,m) as the starting point to traceback to the end point s(0,0). If $S(i,j) = S(i-1, j-1) + s(a_i, b_j)$ then the trajectory is :$(i,j) \rightarrow (i-1, j-1)$.

d. Determine the alignment results
- Notify pairs of DNA as $a_i, b_i$ if the backward path starts from $a_i, b_i$ to the upper left corner.
- Insert a virtual symbol on a vertical sequence and denote it as $(a_i, -)$ if the path is backward horizontally
- Insert a virtual symbol on the horizontal sequence and denote it as $(-, b_i)$ if the backward path is vertical

As examples of known sequences of DNA with sizes 7 and 6 are as follows:
Sequence 1: TCGATTA → length: 7
Sequence 2: CGTGCA → length: 6
Then an initial S value matrix of 8x7 is made, as below

**Tabel 1.** Example of the initial S value matrix

| S(i,j) | | C | G | T | G | C | A |
|---|---|---|---|---|---|---|---|
| | 0 | | | | | | |
| T | | | | | | | |
| C | | | | | | | |
| G | | | | | | | |
| A | | | | | | | |
| T | | | | | | | |
| T | | | | | | | |
| A | | | | | | | |

IOP Conf. Series: Journal of Physics: Conf. Series **1218** (2019) 012031 doi:10.1088/1742-6596/1218/1/012031

For example:
Match score: 5
Mismatch score: -3
Gap score (d): 3

**Tabel 2.** Examples of s substitution matrix results

| $s(a_i, b_i)$ | | C | G | T | G | C | A |
|---|---|---|---|---|---|---|---|
| | 0 | -1 | -2 | -3 | -4 | -5 | -6 |
| T | -1 | -3 | -3 | 5 | -3 | -3 | -3 |
| C | -2 | 5 | -3 | -3 | -3 | 5 | -3 |
| G | -3 | -3 | 5 | -3 | 5 | -3 | -3 |
| A | -4 | -3 | -3 | -3 | -3 | -3 | 5 |
| T | -5 | -3 | -3 | 5 | -3 | -3 | -3 |
| T | -6 | -3 | -3 | 5 | -3 | -3 | -3 |
| A | -7 | -3 | -3 | -3 | -3 | -3 | 5 |

Then the S value matrix is obtained as the following table:

**Tabel 3.** Example of an S value matrix

| | | C | G | T | G | C | A |
|---|---|---|---|---|---|---|---|
| | 0 | -3 | -6 | -9 | -12 | -15 | -18 |
| T | -3 | -3 | -6 | -1 | -4 | -7 | -10 |
| C | -6 | 2 | -1 | -1 | -4 | 1 | -2 |
| G | -9 | -1 | 7 | 4 | 1 | -2 | -2 |
| A | -12 | -4 | 4 | 4 | 1 | -2 | 3 |
| T | -15 | -7 | 1 | 9 | 6 | 3 | 0 |
| T | -18 | -10 | -2 | 6 | 6 | 3 | 0 |
| A | -21 | -13 | -5 | 3 | 3 | 3 | 8 |

In the example above, the traceback step starts from S(7,6)
*Score current* = 8 (matrix score i,j)
*Score diagonal* = 3+5 = 8
*Score left* = 3-3 = 0
*Score up* = 0-3 = -3
Because *score current* = *Score diagonal* = 8, then
i-1 = 7-1 = 6
j-1 = 6-1 = 5
so, the next cell is S(6,5).
For S(6,5):
*Score current* = 3
*Score diagonal* = 6-3 = 3
*Score left* = 6-3 = 3
*Score up* = 3-3 = 0
Because *score current* = *Score diagonal*= 3, then
i-1 = 6-1 = 5
j -1= 5-1=4
then, the next cell is S(5,4).

Traceback step the traceback step is carried out until the last element is S(0,0).

**Tabel 4.** Example of traceback step

| | | C | G | T | G | C | A |
|---|---|---|---|---|---|---|---|
| | 0 | -3 | -6 | -9 | -12 | -15 | -18 |
| T | -3 | -3 | -6 | -1 | -4 | -7 | -10 |
| C | -6 | 2 | -1 | -1 | -4 | 1 | -2 |
| G | -9 | -1 | 7 | 4 | 1 | -2 | -2 |
| A | -12 | -4 | 4 | 4 | 1 | -2 | 3 |
| T | -15 | -7 | 1 | 9 | 6 | 3 | 0 |
| T | -18 | -10 | -2 | 6 | 6 | 3 | 0 |
| A | -21 | -13 | -5 | 3 | 3 | 3 | 8 |

From the example above, the alignment results are obtained as below:

```
Sequence 1:    TCGAT-TA
                ||  |   |
Sequence 2:    -CG-TGCA
```

See from the results of the sequence alignment above, shows that both sequences have mutations in the 7th nucleotide, that is, T in the first sequence changes to C on the second sequence. The score of the alignment of both sequences is 8 and the homology is 50%

## 3. Result and Discussion

Before identifying the location of mutations from two DNA sequences, the process of aligning two DNA sequences is done first using the Needleman-Wunsch algorithm. Here the corona virus DNA sequence data will be used with the following details:

DNA sequence data1: *Avian infectious bronchitis virus (strain D1466) peplomeric protein gene encoding the S1 and S2 subunits, complete cds* with length is 1605 bp ( version X00509.1) year 1989.

```
ATGTGGGCATCGTTACTGTCAGTAGTGACTCTTTTGTTTGCTTTAAGTGAATGTAGTATAGTAGGTGAAAATTACACATACTATTACCAGAGTCAGTTTAGGCCGCCTAATGGCTGGCA
TAAACATGGTGGAGCCTATCTTGTAACCAATGAAACTGACATATCCTATAATGGTGTGTCTTGTACTGTGGGTACAATAAAAGGCGGCATTGTCATTAATGAGAGTGCTATATCTTTT
GTTACAAAAACACCCATTGCTTGGTCAGCCAACGGCGTTTGCACTACATATTGTAATTACTCCAGCTTATATGTGTTTGTTACCCATTGTGGGGGCAGCGGACATACTAGTTGTATTAA
AATACAAATCGCATAGGCGAGATTGTTTTAGGTGTTAAAGACTTTTCTGGTAACTGGATTTATAATCGTACTATAAAGGCTATTGGTCCGTATAGTAAATTTACAGCCTGGCAATGTCT
TGCTAATTTTACCAGTGTGTTTCTAAACGGCAACCTTGTGTATAGTTCTAACTTTACGGAGGATGTTGCAGCGGGTGGTGTTTATGCTAAAAGCGTCAATGGTCTAAAACGTAGAATTA
TGAAGGACACTGATGTTTTGGCATATTTTGTAAATGGCACTGCTGTTGAAGTGATTGTTTGTGATGACAGCCCTAGAGGTAGGTTAGCATGTCAGTATAATACAGGAAATTTTACTGA
TGGGTTATACCCTTTCGTAAGTTACAATGTAGTTAATAATAGTGTTGTTGTTTATGAGGTTATTAGTACTACAACTTATGGTAAACTTAACAACATTACTTTTCATAATGAAACTGGTG
CACCACCTGCAGGTTCTAATGTTGCTAATTTTATTAAATATCAGACGCATGTGGTGCCTGAAGGTTTTGTTAGGCTCAATTTTTCTTTCTTGTCTACTTACAGGTATCAGGAGTCTGATT
TTACTTATGGTTCTTATCATAAGGCTTGTAATTTTAGACTAGAAAGTATTAATAATGGTTTAATGTTTAATACTTTAAGTGTTTCTATTAGCTATGGACCACTTAAGGGTTCTTGTAAGC
AGTCAGTATTTAATCGTAAAGCAACATGCTGTTATGCCTATAAATATCCCACTAATGGGGTTCAAGAGTGTAAGGGTGTTTATAATGGAGAACGCAATACTAAATTTGAATGCGGGCT
TCTTGTATTTATAGACAAGACTGATGGTTCACGCATAATAACTGCAGAAAAACCACCTGTTTATACTACTAATTTTACTAATAATATTGTTGTTGGTAAGTGTGTTAATTATAATATTT
ATGGCAGGTATGGCCAAGGCGTCATTAGTAATATAACTACTGAAGCATTTGGATTTTTACAGGGAGATGGTTTGGTCATCTTGGACACTGCTGGTTCTATAGATATTTTTTCTGTTAAG
GATGGGCCACTCACACATTATTACAAAATTAATCCTTGTAATGATGTAAATCAACAATATGTAGTGTCAGGAGGAAATATAGTTGGTCTTCTCACATCTAGTAATGAGACTGGCTCTA
TTCAGTTAGAAGATCAGTTTTATATTAAACTCACTAATAGCACTCGTAGGCATAGGAGA
```

DNA sequence data 2: *Avian infectious bronchitis virus (strain V1397) peplomeric protein gene encoding the S1 and S2 subunits, complete cds* with length is 1605 bp ( version J02252.1) year 1989

```
ATGTTGGCACAGTTACTGTTAGCAGTGACTCTTTTGTCTGCTTTAGGTGAATGTAGTATAGTAGGTGAAAATTACACATACTATTACCAGAGTCAGTTTAGACCGCCTAATGGCTGGCA
TAAACATGGTGGAGCCTATCTTGTAGTTAATGAAACTGATATATCCTATGATGCTGCGTCTTGTACTGTGGGTACAATAAAAGGCGGCATTGTCATTAATGAGAGTGCTATATCTTTTG
TTACTAAAACACCTATTGCTTGGTCAGCTCAAGGCGTTTGCACTACATATTGTAATTATTCCAGCCTATATGTGTTTGTAACCCATTGTGGGGGCCGTGGACATAATAGTTGTATTATA
AATACAAATCGCATAGGCGAGATTGTTTTAGGTGTTAAATCCTTTTCTGGTAACTGGATTTATAATCGCACTATACAGGCTACTGGTCCGTATAGTAAATTTACAGCCTGGCAATGTCT
TGCTAATTTTACCAGTGTGTTTCTAAATGGCAACCTTGTGTATAGTTCTAACTTTACGGAGGATGTTGCAGCGGCTGGTGTTTATGCTAAAACAGTCAATGGTCTAAAACGTAGAATTA
TGAAGGACACTGATGTTTTGGCATATTTTGTAAATGGCACTGCTGTTGAAGTGATTGTTTGTGATGACAACCCTAAAGGTAGGTTAGCATGTCAGTATAATACAGGAAATTTTACTGA
TGGGTTATACCCTTTCGTAAGTAATAATGTAGTTAATGATAGTGTTGTTGTTTATGATGTTATTAGTACTACAACTTATGGTAAACTTAACAACATTACTTTCCATAATGAAACTAGTG
CACCACCTGCAGGTTCTAATGTTGCTAATTTTATTAAATATCAGACGCATGTGTTGCCTGAAGGTTTTGTTAGGCTTAATTTTTCTTTCTTGTCTACTTACAGGTATCAGGAGTCTGATT
TTACTTATGGTTCTTATCATAAGGCTTGTAATTTTAGACTAGAAAGTATTAATAATGGTTTAATGTTTAATACTTTAAGTGTTTCTATTAGCTATGGACCACTTAAGGGTTCTTGTAAGC
AGTCAGTGTTTAATCATAAAGCAACGTGCTGTTATGCCTATAAATATCCCACTAATGGGGTTCAAGAGTGTAAGGGTGTTTATAATGGAGAACGCAATACTAAATTTGAATGCGGGCT
TCTTGTATTTATAGACAAGACTGATGGTTCACGCATAATAACTGCAGAAAAACCACCTGTTTATACTACTAATTTTACTAATAATATTGTTGTTGGTAAGTGTGTTAATTATAATATTT
ATGGTAGGTATGGCCAAGGCGTCATTAGTAATGTAACTACTGAAGCATTTGGTTTTTTAGAGGGAGATGGTTTGGTCATCTTGGACACTGCTGGTTCTATAGATATTTTTGTTGTTAGG
GATGGTCCATTCACACATTATTACAAGATTAATCCTTGTAATGATGTAAATCAACAATATGTAGTGTCAGGAGGAAATATAGTTGGTCTTCTCACATCTAGTAATGAGACTGGCTCTA
TTCAGTTAGAAGATCAGTTTTATATTAAACTCACTAATAGCACCCGTAGGCATCGGAGA
```

with score:

Match = 5
Mismatch = -3
Gap = -3

The determination of the score above depends on the user and each different score will produce different alignment and homology results.

**Alignment result:**

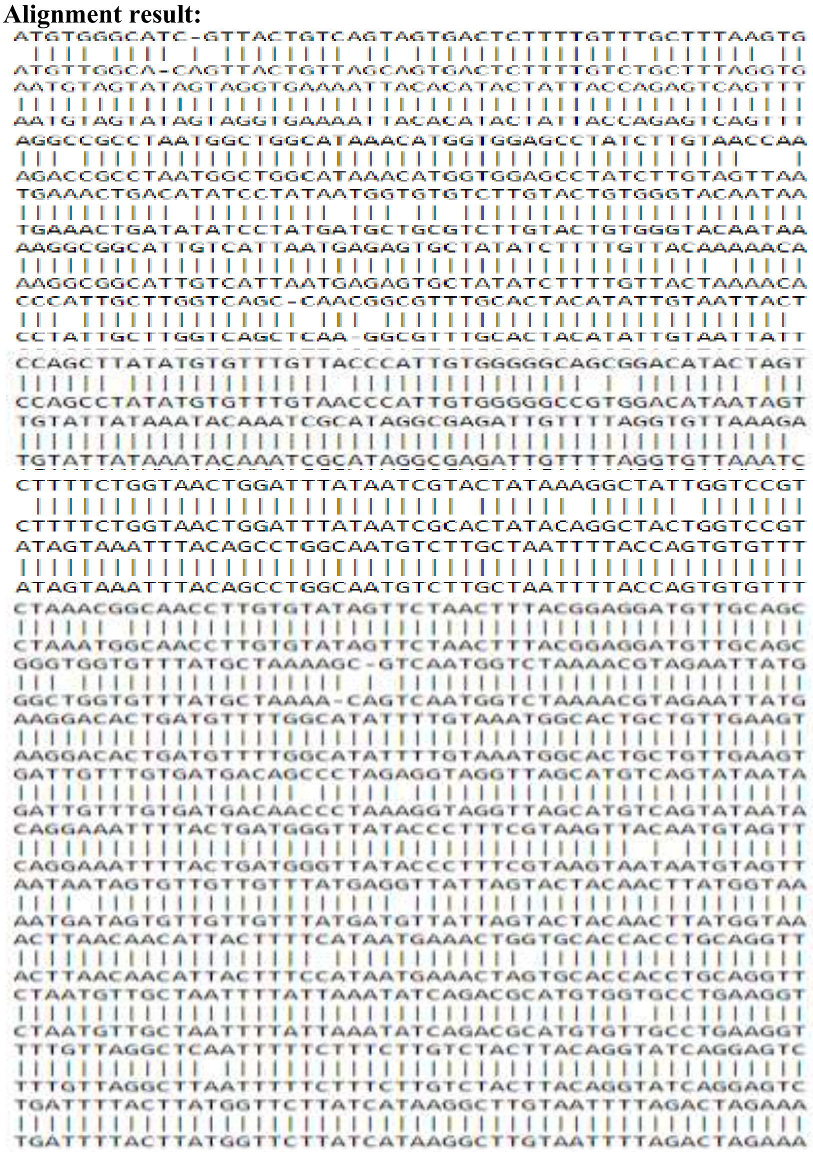

```
GTATTAATAATGGTTTAATGTTTAATACTTTAAGTGTTTCTATTAGCTAT
GTATTAATAATGGTTTAATGTTTAATACTTTAAGTGTTTCTATTAGCTAT
GGACCACTTAAGGGTTCTTGTAAGCAGTCAGTATTTAATCGTAAAGCAAC
GGACCACTTAAGGGTTCTTGTAAGCAGTCAGTGTTTAATCATAAAGCAAC
ATGCTGTTATGCCTATAAATATCCCACTAATGGGGTTCAAGAGTGTAAGG
GTGCTGTTATGCCTATAAATATCCCACTAATGGGGTTCAAGAGTGTAAGG
GTGTTTATAATGGAGAACGCAATACTAAATTTGAATGCGGGCTTCTTGTA
GTGTTTATAATGGAGAACGCAATACTAAATTTGAATGCGGGCTTCTTGTA
TTTATAGACAAGACTGATGGTTCACGCATAATAACTGCAGAAAAACCACC
TTTATAGACAAGACTGATGGTTCACGCATAATAACTGCAGAAAAACCACC
TGTTTATACTACTAATTTTACTAATAATATTGTTGTTGGTAAGTGTGTTA
TGTTTATACTACTAATTTTACTAATAATATTGTTGTTGGTAAGTGTGTTA
ATTATAATATTTATGGCAGGTATGGCCAAGGCGTCATTAGTAATATAACT
ATTATAATATTTATGGTAGGTATGGCCAAGGCGTCATTAGTAATGTAACT
ACTGAAGCATTTGGATTTTTACAGGGAGATGGTTTGGTCATCTTGGACAC
ACTGAAGCATTTGGTTTTTTAGAGGGAGATGGTTTGGTCATCTTGGACAC
TGCTGGTTCTATAGATATTTTT-TCTGTTAAGGATGGGCCACTCACACAT
TGCTGGTTCTATAGATATTTTTGT-TGTTAGGGATGGTCCATTCACACAT
TATTACAAAATTAATCCTTGTAATGATGTAAATCAACAATATGTAGTGTC
TATTACAAGATTAATCCTTGTAATGATGTAAATCAACAATATGTAGTGTC
AGGAGGAAATATAGTTGGTCTTCTCACATCTAGTAATGAGACTGGCTCTA
AGGAGGAAATATAGTTGGTCTTCTCACATCTAGTAATGAGACTGGCTCTA
TTCAGTTAGAAGATCAGTTTTATATTAAACTCACTAATAGCACTCGTAGG
TTCAGTTAGAAGATCAGTTTTATATTAAACTCACTAATAGCACCCGTAGG
CATAGGAGA
CATCGGAGA
```

**Homology:** 96.33312616532007 %
**Score:** 5
**The location of the mutation**
Total of Mutation: 51
Nucleotide to- :

- 5 (changes in nucleotide G to T)
- 21 (changes in nucleotide C to T)
- 24 (changes in nucleotide T to C)
- 39 (changes in nucleotide T to C)
- 47 (changes in nucleotide A to G)
- 103 (changes in nucleotide G to A)
- 146 (changes in nucleotide A to G)
- 147 (changes in nucleotide C to T)
- 148 (changes in nucleotide C to T)
- 160 (changes in nucleotide C to T)
- 170 (changes in nucleotide A to G)
- 174 (changes in nucleotide G to C)
- 177 (changes in nucleotide T to C)
- 244 (changes in nucleotide A to T)
- 253 (changes in nucleotide C to T)
- 299 (changes in nucleotide C to T)
- 306 (changes in nucleotide T to C)
- 320 (changes in nucleotide T to A)
- 336 (changes in nucleotide A to C)
- 338 (changes in nucleotide C to T)

346 (changes in nucleotide C to A)
399 (changes in nucleotide G to T)
400 (changes in nucleotide A to C)
428 (changes in nucleotide T to C)
435 (changes in nucleotide A to C)
442 (changes in nucleotide T to C)
506 (changes in nucleotide C to T)
553 (changes in nucleotide G to C)
668 (changes in nucleotide G to A)
674 (changes in nucleotide G to A)
739 (changes in nucleotide T to A)
741 (changes in nucleotide C to T)
754 (changes in nucleotide A to G)
774 (changes in nucleotide G to T)
819 (changes in nucleotide T to C)
832 (changes in nucleotide G to A)
889 (changes in nucleotide G to T)
912 (changes in nucleotide C to T)
1083 (changes in nucleotide A to G)
1091 (changes in nucleotide G to A)
1101 (changes in nucleotide A to G)
1317 (changes in nucleotide C to T)
1345 (changes in nucleotide A to G)
1365 (changes in nucleotide A to T)
1372 (changes in nucleotide C to G)
1431 (changes in nucleotide A to G)
1438 (changes in nucleotide G to T)
1442 (changes in nucleotide C to T)
1459 (changes in nucleotide A to G)
1594 (changes in nucleotide T to C)
1604 (changes in nucleotide A to C)

Re-testing of 10 Infectious bronchitis virus corona type DNA viruses from 1985 to 1992 with a link as input. This test is done with 3 match values, mismatch and gap, that is:

1. Match= 5, mismatch= -3, and gap= 7
2. Match= 5, mismatch= -3, and gap= 3
3. Match= 9, mismatch= -4, and gap= 4
4. Match= 9, mismatch= -6, and gap= 6
5. Match= 10, mismatch= -6, and gap= 6
6. Match= 14, mismatch= -8, and gap= 8
7. Match= 7, mismatch= -2, and gap= 8

and the results obtained in Figure 3.1, 3.2 and 3.3 are as follows:

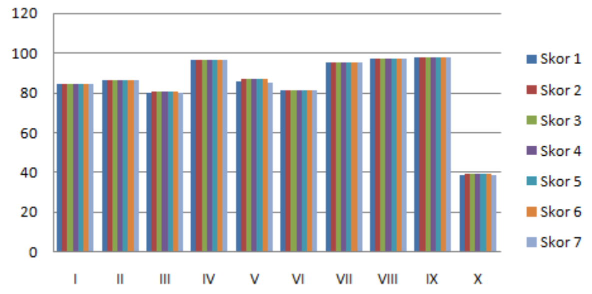

**Figure 3.** Homology level of 7 variations of match values, mismatch, and gap (in percent)

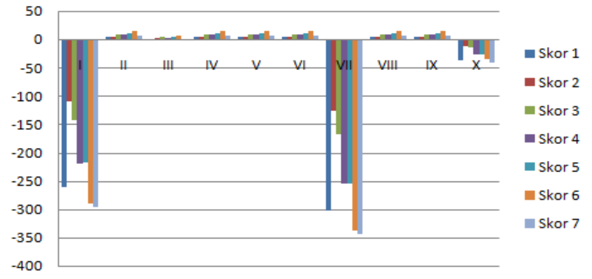

**Figure 4.** Maximum score of 7 variations of match values, mismatch, and gap

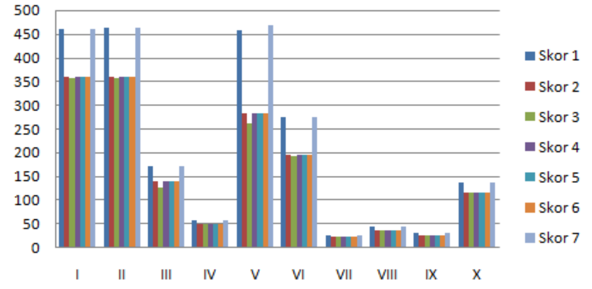

**Figure 5.** Number of Mutations from 7 variations of match values, mismatches, and gaps

Based on experiments conducted on 10 corona virus types infectious bronchitis virus from 1985 to 1 992 with 7 different variations of match, mismatch and gap values, obtained:

1. Based on Figure 3, the results of homology of 10 types of viruses from one time to another over 70 % (meaning that the viral DNA has similarities) but the first type of virus with the last type experien ced a difference of 39% with 117 mutations (on the score to 2 to 6) and 138 (in scores 1 and 7). This shows that the corona virus DNA in the type of infectious bronchitis virus in 1985 and 1992 has und ergone a mutation.
2. The homology level uses 7 variations of match values, different mismatches and gaps produce almo st the same homology level (difference below 1), this shows that the determination of match values, mismatch and gap does not affect the level of homology.
3. Based on Figure 4, 7 out of 10 experiments (2nd, 3rd, 4th, 5th, 6th, 8th, and 9th trials) gave the max imum score at score 6, the score with match = 14, mismatch = -8 and gap = 8. 7 variations of match values, different mismatches and gaps produce different maximum scores. This shows that determin ing the match value, mismatch and gap affect the maximum score.
4. Based on Figure 5, variations in match values, mismatches and gaps 2, 4, 5, and 6 produce the same mutations. However, in variations in match values, mismatches and gaps 1, 3 and 7 produce differen t mutations. This shows that the determination of match values, mismatch and gap affects mutations .

IOP Conf. Series: Journal of Physics: Conf. Series **1218** (2019) 012031 doi:10.1088/1742-6596/1218/1/012031

**Test Validation Algorithm**

To test the truth of the Needleman-Wunsch algorithm, sequence sequencing is done in 2 conditions, namely

1. 2 different sequences but the same length
2. 2 sequences of the same arrangement and length

The following are the results of alignment sequences with the conditions as above:

a. 2 different sequences but the same length as:

Sequence 1: ABCDEFGHIJKLM (length=13)
Sequence 2: NOPQRSTUVWXYZ (length=13)

ABCDEFGHIJKLM

NOPQRSTUVWXYZ

Match: 5
Mismatch: -3
Gap: 3
Homology: 0 %
Score: -3
Total Mutation: 13 (that is, sequence 1 and sequence 2 are different sequences)
Index 1 (change A to N)
Index 2 (change B to O)
Index 3 (change C to P)
Index 4 (change D to Q)
Index 5 (change E to R)
Index 6 (change F to S)
Index 7 (change G to T)
Index 8 (change H to U)
Index 9 (change I to V)
Index 10 (change J to W)
Index 11 (change K to X)
Index 12(change L to Y)
Index 13 (change M to Z)

b. 2 sequences of the same arrangement and length Sequen 1: AAAAAAAAAAAAA (length=13)

Sequen 2: AAAAAAAAAAAAA (length=13)

AAAAAAAAAAAAA
|||||||||||||
AAAAAAAAAAAAA

Match: 5
Mismatch: -3
Gap: 3
Homology: 100 %
Score : 5
Mutation: None (meaning sequence 1 and sequence 2 are the same sequence)

From the two alignments performed, it can be concluded that the Needleman-Wunsch algorithm is correct and can be used to align two sequences.

## 4. Conclusion

Based on the analysis of the results of program testing, it can be concluded that the Needleman-Wunsch algorithm can be applied to identify mutations in the DNA sequence of the corona virus. The test results were carried out on 10 corona virus DNA types of Infectious bronchitis from 1985 to 1992 in sequence. The first type of virus with the last type experienced a difference of 39% and 117 mutations (variation of match, mismatch and gap scores to 2,4,5 and 6) and 138 mutations (variation of match score,

IOP Conf. Series: Journal of Physics: Conf. Series **1218** (2019) 012031 doi:10.1088/1742-6596/1218/1/012031

mismatch and gap to 1 and 7). ) It can also be observed that determining match scores, different mismatches and gaps will produce the same homology but different mutations and maximum scores. This shows that the viral Infectious bronchitis type corona virus DNA in 1985 and 1992 had undergone a mutation (insertion) or deletion (reduction) in the DNA of the virus.
